# Use of a mixed culture strategy to isolate halophilic bacteria with antibacterial and cytotoxic activity from the Manaure solar saltern in Colombia

**DOI:** 10.1186/s12866-017-1136-x

**Published:** 2017-12-08

**Authors:** Natalia Conde-Martínez, Alejandro Acosta-González, Luis E. Díaz, Edisson Tello

**Affiliations:** Bioscience Doctoral Program, Grupo de Investigación en Bioprospección, Faculty of Engineering, Universidad de La Sabana, Campus Puente del Común, Km 7, Autopista Norte de Bogotá, Chía, Colombia

**Keywords:** Mixed cultures, Halophiles, Cytotoxic activity, Antibacterial activity, Solar salterns

## Abstract

**Background:**

Water evaporation in solar salterns creates salinity gradients that promote the adaptation of microbial species to different salinities. This competitive habitat challenges the metabolic capabilities of microorganisms and promotes alterations in their production of secondary metabolites. Thus, solar salterns are a potentially important source of new natural products. In Colombia, the most important and representative solar saltern is located in Manaure (La Guajira) in the north of Colombia. The aim of this study was to develop an alternative screening strategy to select halophilic bacteria as producers of bioactive compounds from mixed microbial cultures rather than individual environmental isolates. Brine and sediment samples from different ponds (across a salinity gradient) were inoculated in seven different culture media to grow bacteria and archaea, allowing for a total of 40 different mixed cultures. An organic extract from each mixed culture was obtained and tested against multidrug resistant pathogens, including *Klebsiella pneumoniae*, vancomycin-resistant *Enterococcus faecium*, methicillin-resistant *Staphylococcus aureus* and *Bacillus subtilis*. In addition, the extracts were tested against two human cancer cell lines, cervical adenocarcinoma (SiHa) and lung carcinoma (A-549).

**Results:**

Twenty-four of the forty extracts from mixed cultures obtained from brine and sediment samples from the Manaure solar saltern showed antibacterial activity against *Bacillus subtilis*. Two extracts, referred to as A1SM3–29 and A1SM3–36, were also active against a methicillin-resistant *Staphylococcus aureus*, with the latter extract also showing slight cytotoxic activity against the assayed human lung cancer cell line. From this mixed culture, nine isolates were cultivated, and their extracts were tested against the same pathogens, resulting in the identification of a *Vibrio* sp. strain (A1SM3–36-8) with antimicrobial activity that was similar to that observed for the mixed culture extract. The extract of this strain was subjected to a bioautography assay, and 3 different fractions exhibited antibacterial activity against methicillin-resistant *Staphylococcus aureus*. Based on the amount obtained for each fraction, F3 was selected to isolate and identify its metabolites. The major compound was identified by NMR and HRMS as 13-*cis*-docosenamide, an amide that has been previously reported to be an antimicrobial and cytotoxic compound.

**Conclusions:**

Our results shows the utility of our strategy in detecting bioactive molecules in initial mixed cultures by biological assays, resulting in the isolation and characterization of *Vibrio* sp. A1SM3–36-8, a halophilic strain with great antibacterial and cytotoxic potential.

**Electronic supplementary material:**

The online version of this article (10.1186/s12866-017-1136-x) contains supplementary material, which is available to authorized users.

## Background

The decrease in the discovery of new metabolites has prompted the exploration of unstudied environments as potential sources of new bioactive compounds. Because marine environments have been a significant source of bioactive metabolites that are useful for drug development [1, 2], solar salterns are emerging as important sites in the search for new metabolites with biological activities. Despite the fact that most of the research on marine organisms has been focused on anticancer agents [3], the rise in antibiotic-resistant pathogens has prompted an increase in research to identify compounds with antimicrobial activity. Such is the case for species of the genus *Salinispora* (obligate halophilic actinobacteria), among other halophilic microorganisms [4–7].

Solar salterns are multi-pond systems in which seawater is pumped through connected ponds to increase salinity by solar evaporation, with final salinities reached that are typically ten times higher than seawater. Salterns are typically found close to coastal areas and are commonly used to precipitate different salts, such as calcium carbonate, calcium sulfate and sodium chloride [8]. These environments have been investigated for their microbial diversity, primarily in ponds with salinities close to the saturation point [6, 9–12]. Halophilic microorganisms are classified according to the optimal salt concentration required for their growth, with species ranging from slightly halophilic (grows in 1–3% salt) to moderately halophilic (grows in 3–15% salt) and extremely halophilic (grows in 15–32% salt) [13]. In Colombia, the Manaure solar saltern, located in the La Guajira region, is the most important and representative solar saltern in the country, followed by the Galerazamba solar saltern in the Atlántico region.

Traditionally, screening for microorganisms with antimicrobial activities is dependent of the isolation of individual strains, a highly time-consuming process compared to the use of mixed cultures, which allows all of the compounds produced by the microorganisms in the mixed culture to be evaluated simultaneously [14]. Thus, the use of mixed cultures has been proposed as an efficient strategy for the selection of microorganisms with potential bioactivities compared to the traditional approach, since more samples can be processed and tested, and more culture conditions can be evaluated. In addition, once the bioactive mixed cultures are selected, the recovery of individual strains is desirable to produce and study the metabolites associated with these activities.

Antibiotic-resistant bacteria are currently a growing global health problem. The WHO has classified carbapenem and 3rd generation cephalosporin-resistant *Klebsiella pneumoniae* as a critical priority for new antibiotics, while vancomycin-resistant *Enterococcus faecium* (VRE) and methicillin-resistant *Staphylococcus aureus* (MRSA) were classified as high priority [15]. In addition, tracheal, bronchial and lung cancers are collectively the third leading cause of cancer-related deaths among Colombian men, while breast and cervical cancers are the most common cancers in women, causing 4.087 registered deaths between 2007 and 2012 in Colombia [16], highlighting the importance of continued research on natural products. The evaluation and characterization of bioactive metabolites is considered a time-consuming practice. However, the use of common analytical techniques, such as high resolution mass spectrometry (HRMS) and nuclear magnetic resonance (NMR) in bioassays, such as bioautography, allows bioactive metabolites to be identified more efficiently [17].

The purpose of this study was to use a mixed culture approach as a strategy to develop a screening-guided isolation method for identifying bacteria that produce bioactive compounds. Ethyl acetate extracts obtained from the mixed cultures isolated from brine and sediment samples from the Manaure Solar Saltern in La Guajira, Colombia, were used to select bioactive mixed cultures for further study. Once microbes from the mixed cultures were isolated and characterized, and their bioactivities were confirmed, a bioautography assay was used to establish which fractions had the bioactive metabolites for subsequent NMR and LC-QTOF-MS analyses. This approach led to the identification of the most abundant metabolite in a bioactive fraction that had activity against MRSA.

## Methods

### Sampling

Brine and sediment samples were collected from 3 different ponds with salinities of 4, 9 and 15% from the Manaure Solar Saltern in the Dealership Big Group Salinas S.A. located in Manaure, La Guajira Colombia (latitude 11°46′32.0″ N, longitude 72°27′27.4″ W). Brine samples were collected in 500 mL sterile glass bottles. The sediment samples were collected in 50 cm long methacrylate cores. All samples were sealed and transported to the lab in a cooler at 4 °C and were processed within 24 h after sampling. The salinity of each pond was determined *in situ* with a hand salt refractometer (Sper Scientific, China), and the pH and temperature were also measured for each pond.

### Growth and enrichment of mixed cultures

Each brine sample was plated on seven different media supplemented with NaCl at a concentration corresponding to the pond from which the sample was collected (4, 9 or 15% [w*/*v]). For each sediment sample, the first 5 cm of the sediment core was added to 5 mL of water (0.5% salinity [w/v]) and mixed by vortexing. The supernatant was plated on the same media used for brine samples at the salinity of the pond where it was collected. The media selected for this work are presented in Table 1, each of which was supplemented with 50 mg/L of cycloheximide (OXOID, Hampshire, England) as an antifungal agent. For solid media, 15 g/L agar was added. All samples and serial dilutions (10^−1^–10^−4^) were plated and incubated at 30 °C for three weeks. The biomass that grew in the first dilution for each media and each salinity was collected and cryopreserved at −80 °C in 20% glycerol. To obtain sufficient biomass, a 200 μL aliquot of each mixed culture was inoculated in 3 mL of the respective media and incubated at 30 °C for 24 h at 150 rpm. After this incubation, the entire enriched culture was transferred to fresh broth (27 mL) and incubated under the same conditions for 15 days. A total of 40 mixed cultures were obtained (Additional file 1).

**Table 1:** Media selected for the growth of halophilic bacteria and archaea

Medium		Observations	References
M1	Halophile Agar I	Halophilic Bacteria	Handbook of Microbiological Media [55]
M2	DSMZ 372	*Halobacterium* spp.	German Collection of Microorganisms and Cell Culture – DSMZ. Leibniz Institute.
M3	*Salinibacter ruber*	Extreme halophilic Bacteria	Antón et al., 2002 [56]
M4	*Salinibacter ruber*	Extreme halophilic Bacteria	Antón et al., 2000 [10]
M5	MCAT	Extreme halophilic Archaea	Pesenti et al., 2008 [57]
M6	Halophilic Actinobacteria	*Nocardiopsis* sp	Jung et al., 2000 [58]
M7	DSMZ 1018	*Haloferax* spp.	German Collection of Microorganisms and Cell Culture – DSMZ. Leibniz Institute

### Extraction and characterization of crude extracts

The whole culture broth of each mixed culture and each isolate was extracted twice with ethyl acetate (1:1) and the organic fraction was subjected to rotary evaporation under vacuum [18]. The obtained extracts were used for testing all biological activities, and uninoculated medium was extracted and used as a control.

### Antibacterial activity

The antibacterial assay was conducted by direct diffusion in an agar plate. One 10 μL drop, corresponding to 150 μg of the extract (resuspended in 20% DMSO) was pipetted on an agar plate that was pre-inoculated with 100 μL of a 0.5 McFarland inoculum of each pathogenic strain and was then incubated at 37 °C for 24 h [19]. To ensure the diffusion of the drop through the agar plate was reproducible and comparable with standard techniques, such as disk diffusion assay [20], the diameter of the zone of inhibition of the drop was measured and compared with that of antimicrobial susceptibility discs (OXOID, Hampshire, England) containing the same quantity of antibiotic (30 μg), showing good results and comparable inhibition zones (Additional file 2). The extracts were tested against multidrug resistant pathogens, including methicillin–resistant *Staphylococcus aureus* (MRSA; ATCC® BAA-44™), vancomycin-resistant *Enterococcus faecium* (VRE; ATCC® 700221™) and *Klebsiella pneumoniae* (ATCC® 700603™). *Bacillus subtilis* (ATCC® 21556™) was used as a control strain due to its sensitivity to a broad spectrum of antibiotics. Chloramphenicol (3 mg/mL) was used as a positive control and was tested using the same procedure. A solution of 20% DMSO was used as a negative control.

### Cytotoxic activity

The crude extracts were tested for cytotoxic activity against two human cancer cell lines and one non-tumor cell line by an MTT cell proliferation assay [21] using Doxorubicin as a positive control [22, 23]. Human cervix epithelial cells (SiHa; ATCC® HTB-35™) and a human lung cancer cell line (A-549; ATCC® CRM-CCL-185™) were grown in Dulbecco’s Modified Eagle’s Medium (DMEM; Sigma-Aldrich Co., Darmstadt, Germany) supplemented with antibiotics (penicillin 120 IU/mL and streptomycin 100 IU/mL; Gibco/Invitrogen, Paisley, UK) and 10% fetal bovine serum (Eurobio, France). Fibroblasts (L929; ATCC® CCL-1™) were incubated in RPMI medium supplemented with 1% (v/v) L-glutamine (Sigma-Aldrich Co., Darmstadt, Germany), 10% (v/v) fetal bovine serum (Eurobio, France), 1% (v/v) penicillin and 1% (v/v) streptomycin (Gibco/Invitrogen, Paisley, UK). L929 was used as non-tumor cell line for toxicity control. The cells were maintained in a humidified atmosphere of 5% CO_2_ at 37 °C.

The cells were grown to approximately 1 × 10^5^ cells per well in 96-well plates and then were incubated with different concentrations of the extracts and were diluted less than 0.5% in DMSO (10, 25, 50, 80 and 100 μg/mL) at 37 °C with 5% CO_2_ for 48 h. Cell viability was measured by an MTT colorimetric assay, and the 50% inhibitory concentration (IC_50_) was calculated [24, 25]. Statistical analysis was performed using GraphPad Prism 6® and Microsoft® Excel 2016. All experiments were repeated at least three times and the results were expressed as the mean values ± standard deviation. IC_50_ values were obtained by nonlinear regression.

### Enrichment, isolation and characterization of bacteria from mixed cultures

The isolation of bacteria from the bioactive mixed culture was performed by subculturing the culture onto agar plates at different dilutions (10^−1^–10^−4^) until an axenic culture was obtained. Isolates were cryopreserved in 1 mL of the corresponding broth containing 20% (*v*/v) glycerol. The isolates were deposited in the Collection of Microorganisms of Universidad de La Sabana (USAB-BIO). Single colonies were morphologically characterized according to their lifting edge, surface, texture, pigmentation and Gram staining. For antimicrobial and cytotoxic assays, all isolates were grown in 30 mL of medium broth and incubated at 30 °C for 15 days, after which the culture were extracted with ethyl acetate as described above. For *Vibrio* sp. A1SM3–36-8, the growth on TCBS (Thiosulfate Citrate Bile Salts Sucrose) agar plates (Oxoid Limited, Hampshire, England) was determined after 24 h at 30 °C, and the antibiotic susceptibility profile was established using commercial antibiotic discs (Oxoid, Hampshire, England). A total of 11 antibiotics were tested, including vancomycin (5 μg), amoxicillin (10 μg), penicillin G (10 μg), chloramphenicol (30 μg), erythromycin (15 μg), ampicillin (10 μg), tetracycline (30 μg), clindamycin (2 μg), cefoxitin (30 μg), nalidixic acid (30 μg) and rifampicin (5 μg) by disk diffusion tests [20]. The susceptibility or resistance was measured by the diameter of the zones of inhibition and were classified according to Bergey’s Manual of Systematics of Archaea and Bacteria for *Vibrio* species [26]. To evaluate growth of isolates at different salinities, 0–20% (w*/*v) NaCl was supplemented in M3 broth and cultures were incubated at 30 °C for 24 h. The growth was expressed as the number of colony forming units (CFU) and the OD_600_ was measured.

### Amplification and sequencing of the 16S rRNA gene

Taxonomic identification was performed by sequencing and analysis of the entire 16S rRNA gene. For amplification, isolated cells were resuspended in sterile DNAse free water. Cells suspensions were frozen using liquid nitrogen for 24 h. The 16S rRNA gene was amplified by PCR using two universal primers, 27F (5′-AGA GTT TGA TCM TGG CTC AG-3′) and 1492R (5′-TAC GGY TAC CTT GTT ACG ACT T-3′). PCR reactions were performed in 0.2 mL microcentrifuge tubes. Each 50 μL PCR reaction contained 2 μL of lysed cells, 1.4 μL of primers, 0.8 μL of dNTPs (25 mmol/L), 5 μL of Taq buffer (10X), 2.8 μL of MgCl_2_ (50 mmol/L) and 0.8 μL of Taq polymerase. The reaction mixtures were placed in a thermocycler with an initial denaturation step at 95 °C for 2 min, followed by 30 cycles at 95 °C for 1 min, annealing at 50 °C for 1 min, and extension at 72 °C for 2 min, with a final extension at 72 °C for 10 min. The PCR products were evaluated by agarose gel electrophoresis in a 1.2% (*w*/*v*) agarose gel run in 0.5X TAE buffer at 90 V/cm for 1 h, with a HyperLadder III marker (500 bp-5 kb) used to assess the molecular weights of the products [11, 27].

PCR products were subjected to Sanger sequencing using four universal primers 27F, 518F (5′-CCA GCA GCC GCG GTA ATA CG-3′), 800R (5′-TAC CAG GGT ATC TAA TCC-3′) and 1492R (MACROGEN, Korea). Data obtained from sequencing were edited, corrected and assembled with CLCbio® software (https://www.qiagenbioinformatics.com/). All 16S rRNA gene sequences were compared against sequences in the SILVA [28] and NCBI databases. For tree reconstruction, the alignments were processed using the ARB software package [29]. All sequences were aligned using the SINA tool (SILVA Incremental Aligner) [28] and were added to the reference dataset SILVA LTPs123_SSU [30, 31] using the ARB software. For the construction of de novo trees, the neighbor-joining algorithm with Jukes-Cantor correction were used [11]. In addition, the similarity matrix was calculated using distance matrix methods with the ARB software. Sequences with identity values greater than 98.7% compared to the type strain sequences were considered to belong to the same species, and sequences with values lower than 94.5% corresponded to different genera [32].

### MALDI-TOF MS analysis

The colonies grown on agar plates were removed using a sterile stick and were spotted over the wells of a 96-well plate. Next, 1 μL of formic acid was aliquoted into each well and, after drying, 1 μL of the matrix solution (2.5 mg/mL of α-cyano-4-hydroxycinnamic acid in 50% acetonitrile, 2.5% trifluoroacetic acid and 47.5% HPLC water) was aliquoted into each well. The mixture was allowed to dry prior to analysis [33]. Mass spectrometry measurements were performed on a Microflex spectrometer LT MALDI-TOF MS (Bruker Daltonics®) and analyzed using Bruker Flex software and a MALDI Biotyper RTC 3.0 (Bruker Daltonics®). The BTS standard (Bacterial Test Standard) *Escherichia coli* DH5α was used for instrument calibration. All spectra were analyzed in a range of 2000 to 20,000 Da. The protein profiles obtained were used to calculate a correlation dendrogram using RTC Biotyper 3.0 software (Bruker Daltonics®).

### Bioautography of a bioactive extract of *Vibrio sp.* A1SM3–36-8

An agar overlay assay [17] was performed to detect the bioactive fractions in the crude extract of *Vibrio* sp. A1SM3–36-8 against MRSA and *B. subtilis*. The 4 × 7 cm TLC plates (ALUGRAM® SIL G/UV_254_, Macherey-Nagel, Germany) were previously sterilized by UV radiation for 30 min. The crude extract was resuspended in ethyl acetate, spotted onto sterile TLC plates and developed in ethyl acetate. The developed plates were air-dried to completely remove the solvent and then were transferred into a sterile petri dish where they were covered with 10 mL of molten and seeded MH agar. One hundred μL of a 1 McFarland inoculum was used per 10 mL of MH agar for each pathogenic strain to fill the petri dish and cover the TLC plate with a thin layer of agar. After solidification, the petri dish with the TLC plate was incubated at 37 °C for 24 h. To visualize the zones of inhibition, the TLC plate was submerged into a 1 mg/mL solution of MTT reagent (Sigma-Aldrich Co., Darmstadt, Germany) for 10 s and incubated at 37 °C for 2 h to assess the dehydrogenase-activity of the microorganism. The dehydrogenase converts tetrazolium salt into purple formazan, allowing the zones of inhibition to be observed as yellow, pallid halos over the purple growth of the pathogens [34]. The retention factor (Rf) values at which the zones of inhibition were observed were calculated. The extract was posteriorly separated by preparative TLC plates. Fifteen mg of the crude extract was spotted onto the preparative TLC plates and developed with ethyl acetate. The fractions matching the Rfs of the bioactive spots were scratched and washed with ethyl acetate and a mixture of ethyl acetate and methanol (95:5).

### Isolation and identification analysis

The fractions were analyzed by NMR experiments, including ^1^H and ^13^C NMR (Bruker Avance 400, 400 MHz for ^1^H and 100 MHz for ^13^C) using CDCl_3_ as solvent. The fractions were analyzed with an Agilent 6520 Accurate-Mass Quadrupole Time-of-Flight LC-QTOF-MS System (Agilent Technologies, Palo Alto, USA) with a reversed phase C18 analytical column (Agilent, Zorbax SB, 5 μm - 4.6 × 150 mm) using a gradient solvent system with A (water and 0.1% trifluoroacetic acid) and B (acetonitrile and 0.1% trifluoroacetic acid), from 20% B at zero minutes to 40% B in 4 min, to 70% B in 10 min and then to 90% B in 27 min. Five microliters of a 1 mg/mL solution of crude extracts were injected and analyzed at a flowrate of 0.5 mL/min in positive ion mode. The instrument was externally calibrated before the runs and the selected mass range was 100–3000 *m/z*.

## Results

### Bioactivity of mixed cultures

**Fig. 1:**
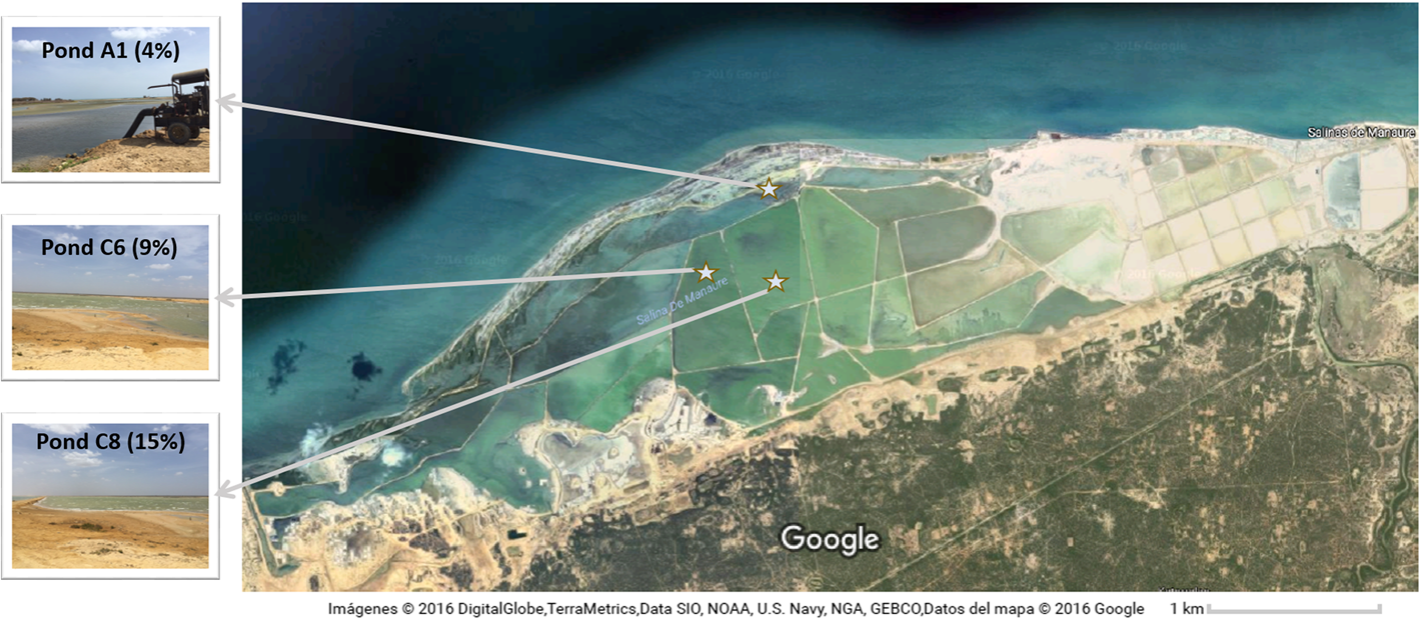
Solar saltern ponds located in the Big Group Salinas Manaure, La Guajira, Colombia

The three ponds selected for sampling had pH values between 7.5–7.8 and a water temperature between 28 and 31 °C. The seawater enters the multi-pond solar saltern system at the A1 pond, which had a measured salinity of 4%. The other two ponds assayed, C6 and C8, are part of the pre-concentrated ponds and had salinities of 9 and 15%, respectively (Fig. 1). After inoculating the three brine (B) and sediment (S) samples (3 each) in seven different media according to the salinity of the ponds (4, 9 or 15%), a total of 40 mixed cultures were obtained (Additional file 1). The ethyl acetate extractions yielded extract amounts of between 0.01 and 0.26 mg/mL.

The results of the antibacterial activity assays showed that 24 of the 40 extracts evaluated had activity against *B. subtilis* (Table 2). Nine extracts were obtained from pond A1 (4% salinity), nine from pond C6 (9% salinity) and 6 from the pond C8, which had the highest salinity (15%). Two extracts, A1SM1–29 and A1SM3–36, which were obtained from mixed microbial cultures of a sediment sample of pond A1 grown on different media (A1SM1–29 on medium M1 and A1SM3–36 on M3) were also active against MRSA (Table 2). The results of the cytotoxic assay showed that two mixed culture extracts displayed activity against a human lung carcinoma cell line (A549), where C8SM3–40, obtained from the sediment sample from pond C8 (15% salinity), had an IC_50_ of 64.60 ± 4.91 μg/mL, and A1SM3–36 had an IC_50_ of 44.90 ± 3.77 μg/mL. None of the extracts evaluated show any toxicity against the non-tumoral cell line L929 (IC_50_ > 100 μg/mL) at the conditions evaluated.

**Table 2:** Antibacterial and cytotoxic activities of the extracts obtained from the 40 mixed cultures against the indicated multidrug resistant pathogens and human cancer cell lines

Extract	Antibacterial Activity				Cytotoxic Activity^a^ (IC_50_ μg/mL)		
	*B. subtilis*	MRSA	VRE	*K. pneumoniae*	A549	SiHa	L929
A1BM2–1	+	–	–	–	> 100	> 100	> 100
A1SM2–2	+	–	–	–	> 100	> 100	> 100
C6BM2–3	+	–	–	–	> 100	> 100	> 100
C6SM2–4	+	–	–	–	> 100	> 100	> 100
C8BM2–5	–	–	–	–	> 100	> 100	> 100
C8SM2–6	+	–	–	–	> 100	> 100	> 100
A1BM4–7	–	–	–	–	> 100	> 100	> 100
A1SM4–8	–	–	–	–	> 100	> 100	> 100
C6BM4–9	–	–	–	–	> 100	> 100	> 100
C6SM4–10	–	–	–	–	> 100	> 100	> 100
C8BM4–11	–	–	–	–	> 100	> 100	> 100
C8SM4–12	–	–	–	–	> 100	> 100	> 100
A1BM7–13	+	–	–	–	> 100	> 100	> 100
A1SM7–14	–	–	–	–	> 100	> 100	> 100
C6BM7–15	+	–	–	–	> 100	> 100	> 100
C6SM7–16	+	–	–	–	> 100	> 100	> 100
C8BM7–17	+	–	–	–	> 100	> 100	> 100
C8SM7–18	–	–	–	–	> 100	> 100	> 100
A1BM5–19	–	–	–	–	> 100	> 100	> 100
A1SM5–20	+	–	–	–	> 100	> 100	> 100
C8BM5–21	+	–	–	–	> 100	> 100	> 100
C8SM5–22	–	–	–	–	> 100	> 100	> 100
A1BM6–23	–	–	–	–	> 100	> 100	> 100
A1SM6–24	+	–	–	–	> 100	> 100	> 100
C6BM6–25	–	–	–	–	> 100	> 100	> 100
C6SM6–26	+	–	–	–	> 100	> 100	> 100
C8BM6–27	+	–	–	–	> 100	> 100	> 100
C8SM6–28	+	–	–	–	> 100	> 100	> 100
A1SM1–29	+	+	–	–	> 100	> 100	> 100
A1BM1–30	+	–	–	–	> 100	> 100	> 100
C6SM1–31	+	–	–	–	> 100	> 100	> 100
C6BM1–32	+	–	–	–	> 100	> 100	> 100
C8SM1–33	–	–	–	–	> 100	> 100	> 100
C8BM1–34	–	–	–	–	> 100	> 100	> 100
A1BM3–35	+	–	–	–	> 100	> 100	> 100
A1SM3–36	+	+	–	–	44.90 ± 3.77	> 100	> 100
C6BM3–37	+	–	–	–	> 100	> 100	> 100
C6SM3–38	+	–	–	–	> 100	> 100	> 100
C8BM3–39	+	–	–	–	> 100	> 100	> 100
C8SM3–40	–	–	–	–	64.60 ± 4.91	> 100	> 100

^a^> 100: IC_50_ value over 100 μg/mL are considered not cytotoxic [59]

### Isolation of microorganisms from mixed cultures and bioactivity assays

Based on the results obtained from the bioactivity assays of the mixed cultures, A1SM1–29 and A1SM3–36 were selected for the isolation of the cultivable strains with similar activities to the mixed cultures by subcultivation of the colonies that grew on agar plates at the plated dilutions until axenic cultures were obtained. A total of 8 isolates for A1SM1–29 and 26 isolates for A1SM3–36 were obtained.

The culture broth extracts from each isolate obtained from A1SM1–29 and A1SM3–36 were tested against *B. subtilis*, MRSA and the A-549 cell line under the same conditions described above. The results showed that the A1SM3–36-8 isolate showed antibacterial activity against MRSA and *B. subtilis* (Additional file 3) and slight activity against the A-549 cell line (30% inhibition at 100 μg/mL).

### Identification of isolates

**Fig. 2:**
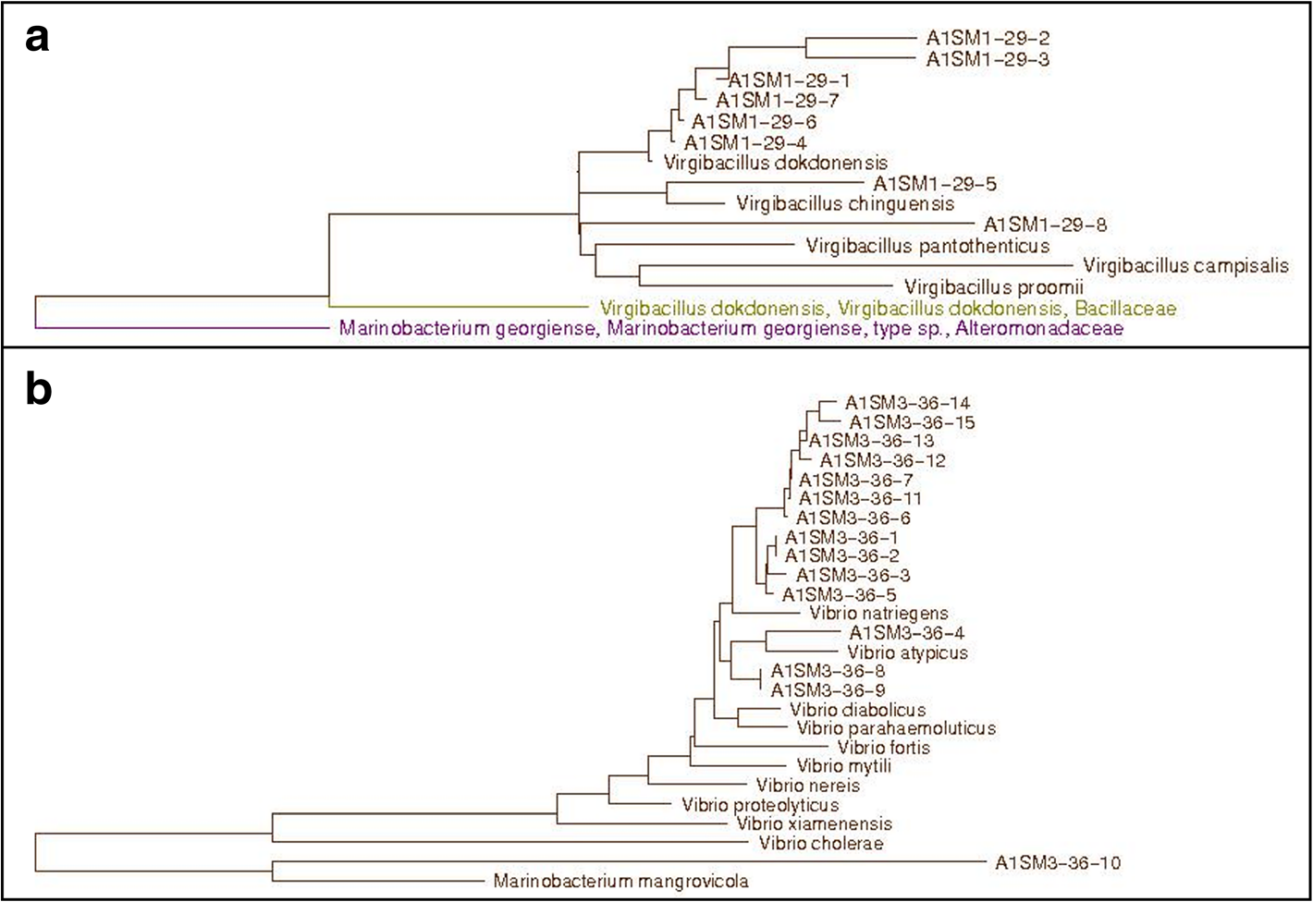
Phylogenetic tree reconstruction based on 16S rRNA genes of the bacterial isolates and their closest relatives. **a** A1SM1–29 isolate and its closest relatives. **b** A1SM3–36 isolate and its closest relatives

The strains isolated from the A1SM1–29 mixed culture were all Gram-positive rods. The 16S rRNA gene of all strains was amplified and sequenced to construct phylogenetic trees by the neighbor-joining method using the aligned sequences. The results led to the determination that all the A1SM1–29 isolates belonged to the genus *Virgibacillus*, having as their closest relatives *V. dokdonensis* (98.8–99.7%) and *V. chiguensis* (98.8–99.6%) (Fig. 2a). The isolate (A1SM1–29-8) was also a close relative of *V. dokdonensi*s and *V. chiguensis* but with lower similarity (98.1 and 97.7%, respectively) than that required to classify it as the same species (98.7%).

For A1SM3–36, the protein profiles obtained by MALDI-TOF MS for each strain were used to calculate a correlation dendrogram in which 9 clades were observed. From this result, 15 of the 26 isolates were selected for amplification and sequencing of the 16S rRNA gene. The phylogenetic tree with the aligned sequences shows that one isolate (A1SM3–36-10) has *Marinobacterium mangrovicola* as the closest relative, with a similarity below the limit of circumscription (96.3%) within the same species [32]. The other isolates (A1SM3–36-1 thru A1SM3–36-15), including isolate A1SM3–36-8, which showed positive results in the bioactivity assays, belonged to the genus *Vibrio*, with *V. natriegens* (99.0–99.3%) and *V. atypicus* (99.3%) as closest relatives (Fig. 2b).

### Characterization of *Vibrio* sp. A1SM3–36-8

Cells were gram-negative and slightly curved rods. After 24 h of incubation, on an M3 agar plate supplemented with 4% (w*/*v) NaCl, circular, creamy, smooth and convex colonies were observed. To establish whether the *Vibrio* sp. A1SM3–36-8 strain was related to *Vibrio atypicus*, catalase activity was determined by the addition of a 1% (v/v) H_2_O_2_ solution over the colonies, and the result was compared to the previously reported activity for this species [35]. The isolate *Vibrio* sp. A1SM3–36-8 showed positive catalase activity that was comparable with the activity observed for *Vibrio harveyi* (ATCC® BAA-1120™), which was used as positive control, suggesting that the isolate A1SM3–36-8 was not related to *Vibrio atypicus*. The antibiotic susceptibility of this isolate was assessed to determine if the isolate had a similar antibiotic profile with other *Vibrio* species (Table 3). According to these results, the susceptibility of the isolate *Vibrio* sp. A1SM3–36-8 to tetracycline, chloramphenicol, and quinolones, such as nalidixic acid, was comparable to the susceptibilities of most environmental *Vibrio* strains, regardless of the species designation [26, 36]. Similarly, the resistance to beta-lactam antibiotics, such as ampicillin and penicillin G, is common for *Vibrio* species [37]. In addition, the growth of the isolate on a selective medium for the genus *Vibrio* (TCBS agar) was evaluated, and yellow, round and smooth colonies were observed after 24 h of incubation, suggesting the presence of *Vibrio* species capable of fermenting sucrose as carbohydrate source. The growth in different salinities showed that *Vibrio* sp. A1SM3–36-8 was a moderate halotolerant bacterium that could grow in salt up to a 9% (w*/*v) NaCl concentration.

**Table 3:** Antibiotic susceptibility profile of the *Vibrio* sp. A1SM3–36-8 isolate

Antibiotic	Susceptibility^a^	Inhibition zone (mm)
Vancomycin	R	–
Amoxicillin	R	–
Penicillin G	R	–
Chloramphenicol	S	28
Erythromycin	I	16
Ampicillin	R	–
Tetracycline	I	19
Clindamycin	R	–
Cefoxitin	I	18
Nalidixic acid	I	20
Rifampicin	S	23

^a^The susceptibility ranges were classified according to Bergey’s Manual of Systematics of Archaea and Bacteria [26]. Resistant (R): Inhibition zones smaller than 11 mm; Intermediate (I): Inhibition zones between 11 and 22 mm determine an intermediate susceptibility; Susceptible (S): Inhibition zones greater than 22 mm determine susceptible strains

### Isolation and identification of a bioactive fraction from *Vibrio* sp. A1SM3–36-8

**Fig. 3:**
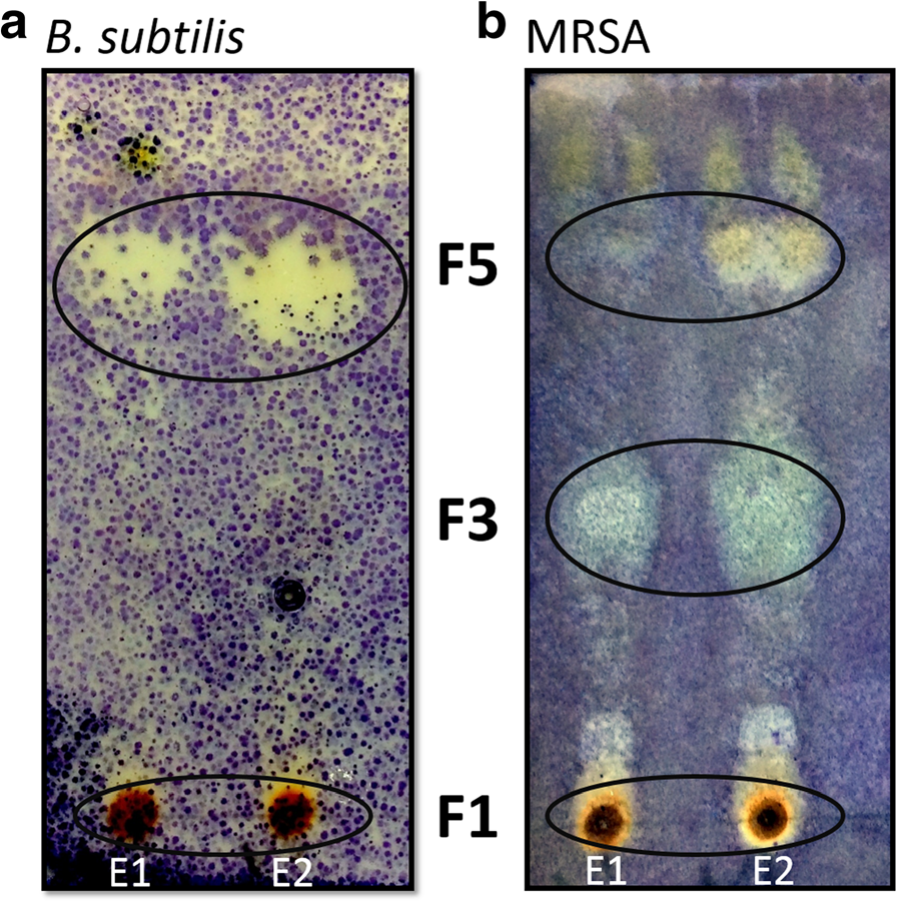
Bioautography assay with two extracts (E1 and E2) from the broth of different cultures of *Vibrio* sp. A1SM3–36-8 spotted on each TLC plate **a** against *B. subtilis,* and **b** against MRSA

From 1 L of *Vibrio* sp. A1SM3–36-8 culture broth, 27 mg of an ethyl acetate extract (2.7%) was obtained, and a bioautography assay was performed to establish at which Rf the zone of inhibition could be detected. From the bioautography assay against MRSA, three zones of inhibition were observed after the TLC plates were assessed with MTT reagent with Rf values of 0.9, 0.4 and 0.1. The bioautography assay against *B. subtilis* showed two zones of inhibition with two of the same Rfs (0.9 and 0.1) (Fig. 3). A preparative TLC was performed to obtain the three primary fractions that corresponded to Rf 0.1 (F1; 2.4 mg), Rf 0.4 (F3; 6.3 mg) and Rf 0.9 (F5; 3.0 mg), which were scratched, extracted and concentrated for further analysis.

**Fig. 4:**
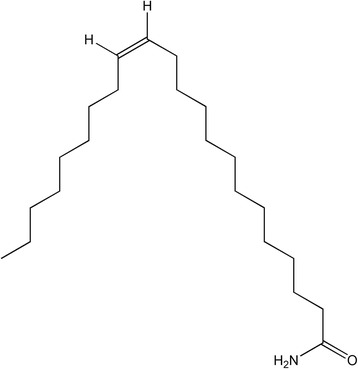
Molecular structure of 13-*cis*-docosenamide

Because of the antibacterial activity exhibited by F3 against MRSA, and the amount of this fraction that was obtained, it was selected for further characterization by NMR and LC-QTOF-MS. The HRMS analysis of the F3 fraction showed the presence of an abundant ion (338.3448 *m/z*) that was consistent with a [M + H]^+^ adduct, and an ion (360.3279 *m/z*) that was consistent with a [M + Na]^+^ adduct, which confirmed an exact mass of 337.3358 uma, corresponding to a molecular formula of C_22_H_43_NO. The ^13^C NMR data of F3 (Table 4) showed one signal located at δ_C_ 175.5 ppm, corresponding to a carbonyl carbon and non-signals for a carbon linked to an oxygen, suggesting the presence of an ester group, allowing to conclude that it could correspond to an amide group according to the nitrogen established by LC-HRMS. Two signals at δ_C_ 129.9 ppm, corresponding to two olefinic methines, suggested the presence of a disubstituted double bond. A group of signals between δ_C_ 36.0 and δ_C_ 22.6 ppm were assigned to 18 methylene carbons. Finally, one signal located at δ_C_ 14.1 ppm was assigned to the unique methyl group observed. The ^1^H NMR spectrum showed one signal at δ_H_ 5.36 ppm (t, 2H, *J* = 4.7 Hz), corresponding to two identical olefinic protons, suggesting a *cis* coupling between them. One triplet methylene located at δ_H_ 2.22 ppm (t, 2H, *J* = 8 Hz) was assigned to the methylene attached to the carbonyl group. The signal at δ_H_ 2.01 ppm (dd, 4H, *J* = 12.1, 6.4 Hz) corresponded to the two methylenes adjacent to the double bond. A multiplet signal located at δ_H_ 1.63 ppm (m, 2H) was assigned to the methylene at beta position to the carbonyl group. A broad signal at δ_H_ 1.26 ppm (br s, 28H) corresponded to the methylene groups associated with an aliphatic chain. The last signal was one triplet at δ_H_ 0.88 (t, 3H, *J* = 6.7 Hz), corresponding to the methyl group at the end of the aliphatic chain (Additional file 4). These data allowed for the identification of a lineal amide with 22 carbons and one unsaturation. Additionally, a database searches was performed in the Spectral Database for Organic Compounds (SDBS, http://sdbs.db.aist.go.jp) and in METLIN (http://metlin.scripps.edu), which confirmed that the spectral information obtained for this compound corresponds to 13-*cis*-docosenamide (Fig. 4).

**Table 4:** ^1^H and ^13^C NMR data for the major compound in the F3 fraction

Position	δ_C_, mult	δ_H_, mult (J in Hz)
1	175.5, s	–
2	35.9, t	2.22, t (8.0)
3	25.5, t	1.63, m
4	29.2, t	1.26, br s
5	29.3, t	
6, 7, 8	29.5, t	
9	29.5, t	
10	29.6, t	
11	29.6, t	
16	29.7, t	
17,18	29.8, t	
19	29.5, t	
20	31.9, t	
21	22,7, t	
12,15	27.2, t	2.01, dd (12.1, 6.4)
13	129.9, d	5.36, t (4.7)
14	129.9, d	
22	14.1, q	0.88, t (6.7)

## Discussion

The strategy of using mixed cultures and subsequent isolation led to the development of a guided isolation method for the identification of bacteria producing antibacterial and cytotoxic metabolites. Two mixed cultures were selected due to their bioactivity, both of which were isolated from the same A1 pond sediment sample (4% salinity). After identifying isolates from each culture, it was evident that the medium in which the sample was inoculated influenced the growth of species from two different genera. *Virgibacillus* and *Vibrio* species were isolated using M1 and M3 media, respectively. The M1 medium had an approximately 6-fold higher amount of nutrients relative to that in the M3 medium, with yeast extract as the primary nutrient source, which has been reported to be used for the isolation of *Virgibacillus* species [38]. Furthermore, the majority of isolated and identified strains were γ-Proteobacteria, consistent with the fact that this class is the most frequently isolated group from marine environments when using plating techniques [39], and the genera *Virgibacillus* and *Vibrio* are largely recognized as halophilic [40, 41].

Using the strategy proposed in this study, the strain responsible for the observed activity in one of the bioactive mixed cultures (A1SM3–36) was isolated. Most of the isolates obtained from A1SM3–36 showed high similarity to *Vibrio natriegens*, a fast-growing marine bacterium associated with the sponge *Stylotella* sp. [42, 43], and this bacterium also has been isolated from three different salt springs in the Boyacá and Cundinamarca regions of Colombia [44], demonstrating the affinity of *Vibrio* species for different saline environments. This species has been reported to be active against *Pseudomonas aeruginosa*, *S. aureus* and *E. coli* and toxic against *Artemia salina*, with an LC_50_ of 156.68 μg/mL [43]. The similarity of the isolates to *V. natriegens* may be due to their rapid growth in minimal media supplemented with glucose, similar to the M3 medium used in this work and as was previously demonstrated by Lee, Gold, and Khalil [45].

In the case of A1SM3–36, the isolation of a bioactive halophilic bacterium through the use of the mixed culture strategy was successful. This approach facilitated the later identification of isolates with bioactivities of interest, in contrast to the large number of isolates that would likely have been obtained if the conventional isolation methods had been used. For instance, in this study, seven growth media, three salinities and two types of samples (brine and sediment) were analyzed, which could have led to a large number of isolates and a time-consuming screening process needed to establish the biological activity of each isolate. Our strategy led to the isolation and characterization of the halophilic bacterium *Vibrio* sp. A1SM3–36-8, which showed great antibacterial activity potential against the multidrug resistant pathogen MRSA and yielded good results for cytotoxic activity against the human lung cancer cell line A-549. In addition, a bioautography assay led to the identification of the bioactive Rfs, which allowed for the selective isolation and partial purification of 13-*cis*-docosenamide. This was achieved in both a practical and effective manner, considering that the amount of the F3 fraction obtained was enough for spectroscopic and spectrometric analysis, in contrast to the amounts obtained from F1 and F5, bioactive fractions from the same extract.

The amide 13-*cis*-docosenamide was previously isolated from the halophilic *Bacillus* sp. BS3, exhibiting biosurfactant activity and potential antimicrobial activity against *E. coli*, *S. aureus*, *P. aeruginosa* and *Salmonella typhi* [46]. It was also identified as a common compound produced by 18 different halophilic bacterial isolates of the genera *Halobacillus*, *Sanilivibrio*, *Oceanobacillus*, *Salinicoccus* and *Thalassobacillus*, which were isolated from a South African Saltpan [47]. In a different study, a 6-*cis*-docosenamide isomer was isolated from *Asimina parviflora* fruit, and its cytotoxic activity against the cell line A-549 was observed, showing a 10-fold greater activity than that observed in this study [48]. However, this difference could be explained because, in our study, the cytotoxic activity of the crude extract was evaluated and not the pure compound. In addition, it is also important to consider the difference in bioactivities between structural isomers.

For A1SM1–29, the obtained isolates were related to *Virgibacillus dokdonensis* and *Virgibacillus chiguensis*, both of which were previously isolated from marine environments. *V. dokdonensis* was isolated from seawater near a Korean island [49] and *V. chiguensis* was isolated from sediment from a commercial saltern in Taiwan [50] using the standard dilution plating method with marine and DSM 372 agar supplemented with 3% and 5% (w*/*v) NaCl, respectively, consistent with the salinity used in our study to culture samples taken from the A1 pond, such as A1SM1–29. *V. dokdonensis* VIT P14 has shown promise in a wide range of applications [51]. As far as we know, there has been no report on the biological activity or industrial applications of *V. chiguensis*. The lack of observed activity for the A1SM1–29 isolates could be explained by two different rationales. The first rationale is that the production of the bioactive metabolites could be influenced by the interactions between the microorganisms co-existing in the culture, as was published in recent study in which the mixed culture or a co-culture was used as an excellent strategy to induce the production of new metabolites in microorganisms grown under laboratory conditions [52, 53]. A metabolomic approach could be useful to compare the metabolic profiles of each strain when grown individually or in a mixed culture to establish which metabolites are induced by the interactions among strains. Glionitrin A, a diketopiperazine disulfide, is produced during the co-culture of a *Sphingomonas* bacterial strain and *Aspergillus fumigatus* but was not detected in the monoculture. This compound displayed an antibiotic activity against MRSA and cytotoxic activity against four human cancer cell lines, HCT-116, A-549, AGS and DU145, suggesting that microbial competition can result in the production of novel chemical structures that could be potential drug candidates [54]. The second rationale is related to the fact that the strain responsible for the bioactivity in A1SM1–29 could not be isolated with the subcultivation techniques used in this study, in contrast to the results obtained for the A1SM3–36 mixed culture, from which the bioactive strain *Vibrio* sp. A1SM3–36-8 was obtained. A metagenomic approach could be used to establish if all species present in the mixed cultures were isolated to develop a selective strategy for their cultivation.

## Conclusion

In this study, we used a strategy in which bioactive molecules in initial mixed cultures were detected by biological assays to guide the screening of bioactive isolates of interest, which led to the isolation and characterization of a *Vibrio* sp. (strain A1SM3–36-8) with antibacterial and cytotoxic potential. However, as isolates obtained from the mixed culture A1SM1–29 did not show biological activity when grown individually, further research is needed to establish whether the production of bioactive metabolites was regulated by the interaction between the strains of this mixed culture or whether the isolation of the responsible strain is problematic. These results highlight that the strategy of using mixed cultures is an efficient alternative to identify cultures of microorganisms with pharmacological applications.

In addition, NMR and LC-QTOF-MS analyses led to identification of the major compound (13-*cis*-docosenamide) in the F3 bioactive fraction, which may be the compound responsible for the antibacterial activity against MRSA observed for the A1SM3–36 extract. The two additional fractions that displayed antibacterial activity could not be characterized due to the low extraction yield. However, this result confirmed the potential of *Vibrio* sp. A1SM3–36-8 to produce several compounds with antimicrobial activity. One future perspective to the approach described in this study is the implementation of a wider panel of bioassays, which could allow for bioassay-guided isolation of new or known bioactive metabolites with new bioactivities.

## Additional files

Additional file 1 Table S1. Mixed cultures obtained from brine and sediment samples from the Manaure solar saltern and extraction yield with ethyl acetate. (DOCX 16 kb)

Additional file 2: Figure S1. Antibacterial assay by direct diffusion in an agar plate. Comparison of the inhibition zones of a 10 μL drop with 30 μg of chloramphenicol with the inhibition diameter of antimicrobial susceptibility discs (OXOID, Hampshire, England) on an agar plate that was pre-inoculated with a 0.5 McFarland MRSA strain inoculum and grown at 37 °C for 24 h. (DOCX 2211 kb)

Additional file 3: Figure S2. Antibacterial activity of the A1SM3–36-8 isolate extract against (a) MRSA and (b) *B. subtilis* in duplicate. The clear regions are the zones of growth inhibition caused by an aliquot containing 150 μg of the extract in 20% DMSO. (DOCX 592 kb)

Additional file 4: Nuclear magnetic resonance spectra of the F3 fraction. (DOCX 107 kb)

## Abbreviations

DMSO: Dimethyl sulfoxide
HRMS: High resolution mass spectrometry
LC-QTOF-MS: Liquid chromatography quadrupole time-of-flight mass spectrometry
MH: Müller Hinton
MRSA: methicillin-resistant *Staphylococcus aureus*
MTT: 3-(4,5-dimethylthiazol-2-yl)-2,5-diphenyltetrazolium bromide
NMR: Nuclear Magnetic Resonance
TLC: Thin Layer Chromatography
USAB-BIO: Collection of Microorganisms of Universidad de La Sabana
VRE: vancomycin-resistant *Enterococcus faecium*
