# Supplementary material for: Use of a mixed culture strategy to isolate halophilic bacteria with antibacterial and cytotoxic activity from the Manaure solar saltern in Colombia
Source: BMC Microbiol. 2017 Dec 8;17:230. doi: 10.1186/s12866-017-1136-x (PMC5721385; doi:10.1186/s12866-017-1136-x)
Supplement: Supplementary file 1 — Mixed cultures obtained from brine and sediment samples from the Manaure solar saltern and extraction yield with ethyl acetate. (DOCX 16 kb) [file 12866_2017_1136_MOESM1_ESM.docx]

**Table S1.** Mixed cultures obtained from brine and sediment samples from the Manaure solar saltern and extraction yield with ethyl acetate.

| Mixed Culture^*^ | Salinity | Extraction Yield  mg/mL |
| --- | --- | --- |
|  |  |  |
| A1BM2-1 | 4% | 0.15 |
| A1SM2-2 | 4% | 0.16 |
| C6BM2-3 | 9% | 0.09 |
| C6SM2-4 | 9% | 0.08 |
| C8BM2-5 | 15% | 0.13 |
| C8SM2-6 | 15% | 0.11 |
| A1BM4-7 | 4% | 0.03 |
| A1SM4-8 | 4% | 0.03 |
| C6BM4-9 | 9% | 0.03 |
| C6SM4-10 | 9% | 0.06 |
| C8BM4-11 | 15% | 0.03 |
| C8SM4-12 | 15% | 0.01 |
| A1BM7-13 | 4% | 0.09 |
| A1SM7-14 | 4% | 0.07 |
| C6BM7-15 | 9% | 0.11 |
| C6SM7-16 | 9% | 0.10 |
| C8BM7-17 | 15% | 0.11 |
| C8SM7-18 | 15% | 0.08 |
| A1BM5-19 | 4% | 0.07 |
| A1SM5-20 | 4% | 0.08 |
| C8BM5-21 | 15% | 0.09 |
| C8SM5-22 | 15% | 0.07 |
| A1BM6-23 | 4% | 0.08 |
| A1SM6-24 | 4% | 0.13 |
| C6BM6-25 | 9% | 0.06 |
| C6SM6-26 | 9% | 0.08 |
| C8BM6-27 | 15% | 0.08 |
| C8SM6-28 | 15% | 0.08 |
| A1SM1-29 | 4% | 0.26 |
| A1BM1-30 | 4% | 0.08 |
| C6SM1-31 | 9% | 0.11 |
| C6BM1-32 | 9% | 0.09 |
| C8SM1-33 | 15% | 0.11 |
| C8BM1-34 | 15% | 0.09 |
| A1BM3-35 | 4% | 0.02 |
| A1SM3-36 | 4% | 0.06 |
| C6BM3-37 | 9% | 0.02 |
| C6SM3-38 | 9% | 0.05 |
| C8BM3-39 | 15% | 0.06 |
| C8SM3-40 | 15% | 0.03 |

* The mixed cultures were named by the corresponding pond of isolation (A1 – C6 – C8); B or S for Brine or Sediment sample; the medium in which the sample was grown initially (M1 to M7, see Table 1) and a consecutive number from 1 to 40, e.g. A1BM2-1; was the mixed culture obtained from the brine sample of A1 pond and grown in M2 medium.
