# Supplementary material for: Use of a mixed culture strategy to isolate halophilic bacteria with antibacterial and cytotoxic activity from the Manaure solar saltern in Colombia
Source: BMC Microbiol. 2017 Dec 8;17:230. doi: 10.1186/s12866-017-1136-x (PMC5721385; doi:10.1186/s12866-017-1136-x)
Supplement: Supplementary file 2 — Antibacterial assay by direct diffusion in an agar plate. Comparison of the inhibition zones of a 10 μL drop with 30 μg of chloramphenicol with the inhibition diameter of antimicrobial susceptibility discs (OXOID, Hampshire, England) on an agar plate that was pre-inoculated with a 0.5 McFarland MRSA strain inoculum and grown at 37 °C for 24 h. (DOCX 2211 kb) [file 12866_2017_1136_MOESM2_ESM.docx]

**Additional file 2**

**Figure S1.** Antibacterial assay by direct diffusion in an agar plate. Comparison of the inhibition zones of a 10 µL drop with 30 µg of chloramphenicol with the inhibition diameter of antimicrobial susceptibility discs (OXOID, Hampshire, England) on an agar plate that was pre-inoculated with a 0.5 McFarland MRSA strain inoculum and grown at 37°C for 24 h.


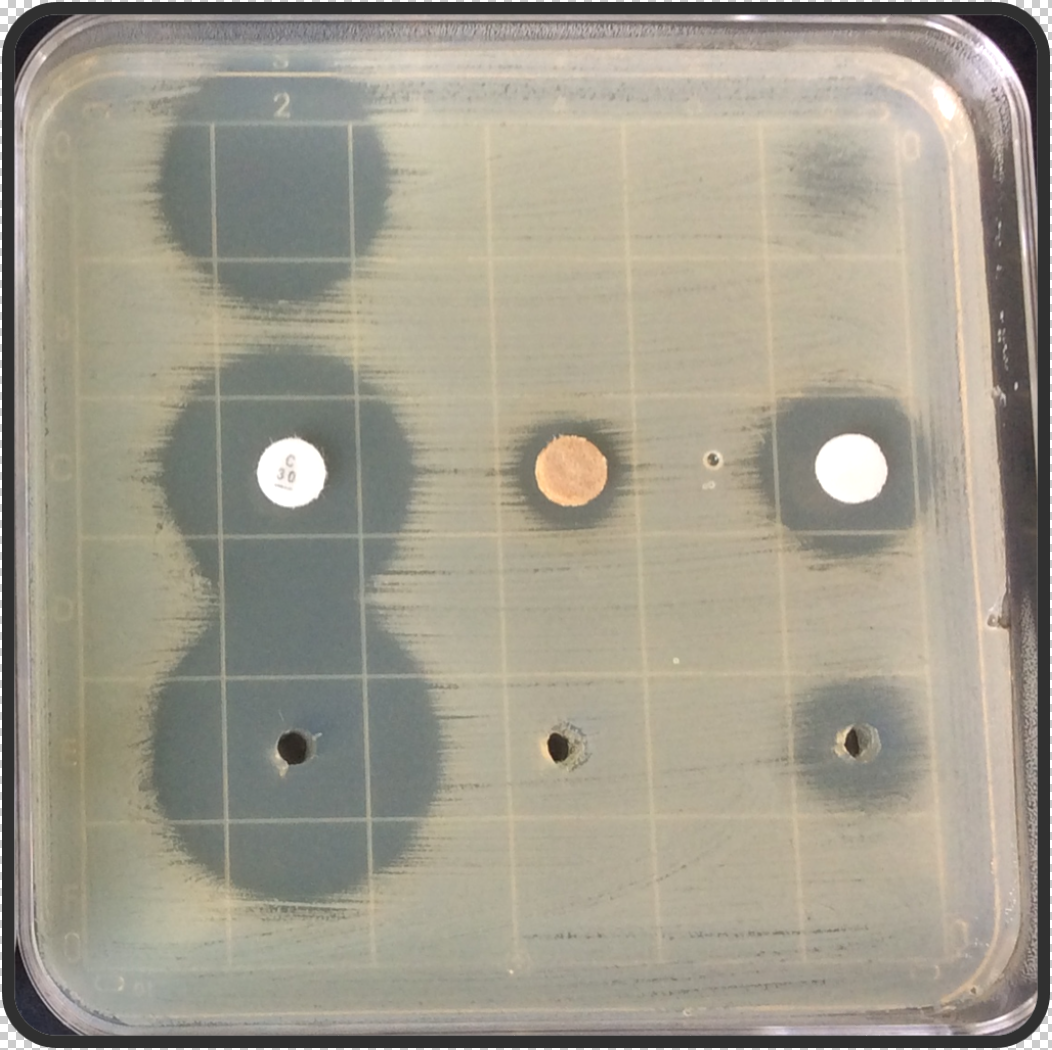


**10 µl drop (30 µg of Chloramphenicol)**
