# Supplementary material for: Use of a mixed culture strategy to isolate halophilic bacteria with antibacterial and cytotoxic activity from the Manaure solar saltern in Colombia
Source: BMC Microbiol. 2017 Dec 8;17:230. doi: 10.1186/s12866-017-1136-x (PMC5721385; doi:10.1186/s12866-017-1136-x)
Supplement: Supplementary file 3 — Antibacterial activity of the A1SM3–36-8 isolate extract against (a) MRSA and (b) B. subtilis in duplicate. The clear regions are the zones of growth inhibition caused by an aliquot containing 150 μg of the extract in 20% DMSO. (DOCX 592 kb) [file 12866_2017_1136_MOESM3_ESM.docx]

**Additional file 3**

**Figure S2.** Antibacterial activity of the A1SM3-36-8 isolate extract against (a) MRSA and (b) B. subtilis in duplicate. The clear regions are the zones of growth inhibition caused by an aliquot containing 150 µg of the extract in 20% DMSO.


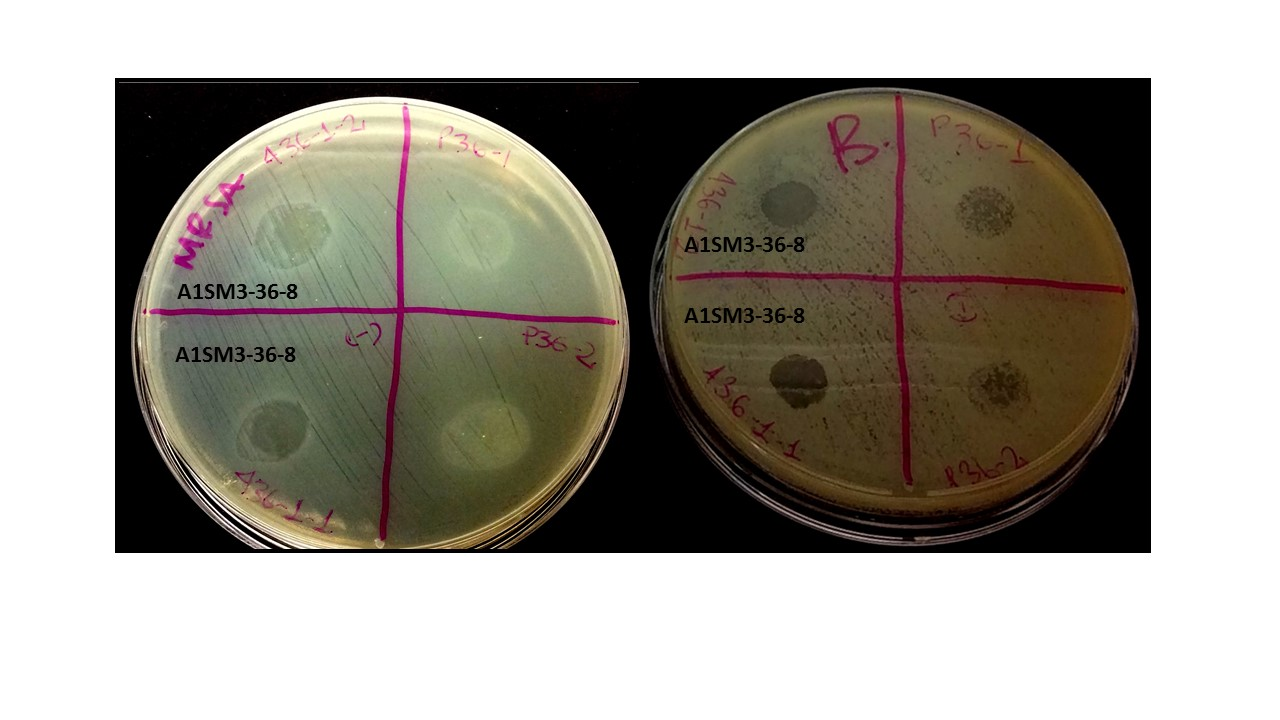


**(a)**

**(b)**
