# Supplementary material for: Use of a mixed culture strategy to isolate halophilic bacteria with antibacterial and cytotoxic activity from the Manaure solar saltern in Colombia
Source: BMC Microbiol. 2017 Dec 8;17:230. doi: 10.1186/s12866-017-1136-x (PMC5721385; doi:10.1186/s12866-017-1136-x)
Supplement: Supplementary file 4 — Nuclear magnetic resonance spectra of the F3 fraction. (DOCX 107 kb) [file 12866_2017_1136_MOESM4_ESM.docx]

^13^C (APT) NMR Spectrum of the F3 fraction (13-*cis*-docosenamide)

^1^H NMR Spectrum of the F3 fraction (13-*cis*-docosenamide)
